# Supplementary material for: Content-rich biological network constructed by mining PubMed abstracts
Source: BMC Bioinformatics. 2004 Oct 8;5:147. doi: 10.1186/1471-2105-5-147 (PMC528731; doi:10.1186/1471-2105-5-147)
Supplement: Additional File 2 — The original results of the above study (non-essential files are deleted to keep the file size under the limit set by BMC bioinformatics). [file 1471-2105-5-147-S2.bz2 › chilibotAdditionalFile2/dip10/252ID7809159E1010/html/CDC2_CKS1.html]

 


 **CDC2** and **CKS1** 
  
Found 40 abstracts in PubMed, retrieved 10.  
 

 What does Google say? 
 PDF only 
| .edu only 

---

**Interactive relationship** (e.g. stimulation, inhibition, etc)

**Neutral relationship**- All proteins of this family have Cdk  [ **CDC2** ]  binding and anion binding sites, but only mammalian  **Cks1**  binds to Skp2 and promotes the association of Skp2 with p27 phosphorylated on Thr 187.  Ref: 12140288 J Biol Chem, 2002
- This activity, which can be independent of Cdk  [ **CDC2** ]  binding, links Cks  [ **CKS1** ]  to positive growth control pathways regulating the G1 S transition and to cancer.  Ref: 11516665 Curr Biol, 2001
- The interaction of Skp2 with the substrate is further strengthened by the association of the Cdk  [ **CDC2** ]  binding site of  **Cks1**  with Cdk2 cyclin E, to which phosphorylated p27 is bound.  Ref: 12140288 J Biol Chem, 2002
- The Cks  [ **CKS1** ]  Suc1 proteins associate with CDK  [ **CDC2** ]  cyclin complexes, but their precise function s is not well defined.  Ref: 11463388 Mol Cell, 2001
- We have purified, from starfish oocytes, a 15 kDa protein, p15 CDK  [ **CDC2** ]  BP, which cross reacts with anti Cks  [ **CKS1** ]  antibodies L.  Ref: 12007796 Biochim Biophys Acta, 2002
- The suc1 Cks  [ **CKS1** ]  proteins are well conserved regulatory components of cyclin dependent kinases 1 and 2 CDK1  [ **CDC2** ]  2.  Ref: 12007796 Biochim Biophys Acta, 2002
- All three binding sites of  **Cks1**  are required for p27 ubiquitin ligation and for the association of Skp2 with Cdk  [ **CDC2** ]  bound, Thr 187 phosphorylated p27.  Ref: 12140288 J Biol Chem, 2002
- Assembly of protein complexes with cyclin Cdk  [ **CDC2** ]  is noted and the capacity of the cyclin dependent kinase subunit Cks  [ **CKS1** ] , in such complex, to extend the range of Cdk  [ **CDC2** ]  substrates is documented and discussed in terms of cell cycle regulation.  Ref: 11732181 Protoplasma, 2001
- **Cks1** , a subunit of cyclin dependent kinases, has now been identified as an essential cofactor in the ubiquitination of the Cdk  [ **CDC2** ]  inhibitor p27 by the SCF Skp2 ubiquitin ligase.  Ref: 11516665 Curr Biol, 2001
- ... overexpression of cdc28 protein kinase 1  [ **CKS1** ]   **CKS1**  blocks the inhibition of the cyclin D1 CDK4 complex by the CDK  [ **CDC2** ]  inhibitor p27 Kip1.  Ref: 12605034 Diagn Mol Pathol, 2003

**Non-interactive relationship** (e.g. studied together, co-existance, homology, etc.)

- This latter process requires the Skp2 and anion binding sites of  **Cks1** , but not its Cdk  [ **CDC2** ]  binding site.  Ref: 12140288 J Biol Chem, 2002
- Human CDK  [ **CDC2** ]  subunit 1  **Cks1**  and S phase kinase associated protein 2 Skp2 are components of the SCF Skp2 complex, which acts as a ubiquitin ligase for p27 Kip1.  Ref: 12670508 Biochem Biophys Res Commun, 2003
- Here we demonstrate that  **Cks1**  directs the ubiquitin mediated proteolysis of the CDK  [ **CDC2** ]  bound substrate p27Kip1 by the protein ubiquitin ligase E3 SCF Skp2.  Ref: 11463388 Mol Cell, 2001
- Despite the high degree of homology between the nine known CDKs, only CDK1  [ **CDC2** ] , CDK2 and, to a lesser extent, CDK3 are able to bind to the suc1 Cks  [ **CKS1** ]  proteins.  Ref: 12007796 Biochim Biophys Acta, 2002
- A CDK  [ **CDC2** ]  independent function of mammalian  **Cks1**  targeting of SCF Skp2 to the CDK  [ **CDC2** ]  inhibitor p27Kip1.  Ref: 11463388 Mol Cell, 2001
- We generated mice lacking Cks2, one of two mammalian homologs of the yeast Cdk1  [ **CDC2** ]  binding proteins, Suc1 and  **Cks1** , and found them to be viable but sterile in both sexes.  Ref: 12714746 Science, 2003
